# Oxidative Stress and Dementia in Alzheimer's Patients: Effects of Synbiotic Supplementation

**DOI:** 10.1155/2020/2638703

**Published:** 2020-01-13

**Authors:** Alyne Mendonça Marques Ton, Bianca Prandi Campagnaro, Gisela Aleixo Alves, Rafaela Aires, Larissa Zambom Côco, Clarisse Maximo Arpini, Trícia Guerra e Oliveira, Manuel Campos-Toimil, Silvana Santos Meyrelles, Thiago Melo Costa Pereira, Elisardo Corral Vasquez

**Affiliations:** ^1^Laboratory of Translational Physiology and Pharmacology, Pharmaceutical Sciences Graduate Program, Vila Velha University, Vila Velha, Espírito Santo, Brazil; ^2^Laboratory of Translational Physiology, Physiological Sciences Graduate Program, Federal University of Espírito Santo, Vitória, Espírito Santo, Brazil; ^3^Pharmacology of Chronic Diseases (CDPHARMA), Molecular Medicine and Chronic Diseases Research Centre (CIMUS), University of Santiago de Compostela, Santiago de Compostela, Spain; ^4^Federal Institute of Education, Science and Technology (IFES), Vila Velha, Espírito Santo, Brazil

## Abstract

**Background:**

Alzheimer's disease (AD) is the most common cause of dementia in elderly patients. Recently, several studies have shown that inflammation and oxidative stress precede the cardinal neuropathological manifestations of AD. In view of the proven antioxidant effects of probiotics, we proposed that continuous dietary supplementation with milk fermented with kefir grains might improve cognitive and metabolic and/or cellular disorders in the AD patients.

**Methods:**

This study was designed as an uncontrolled clinical investigation to test the effects of probiotic-fermented milk supplementation (2 mL/kg/daily) for 90 days in AD patients exhibiting cognitive deficit. Cognitive assessment, cytokine expression, systemic oxidative stress levels, and blood cell damage biomarkers were evaluated before (T0) and after (T90) kefir synbiotic supplementation.

**Results:**

When the patients were challenged to solve 8 classical tests, the majority exhibit a marked improvement in memory, visual-spatial/abstraction abilities, and executive/language functions. At the end of the treatment, the cytometric analysis showed an absolute/relative decrease in several cytokine markers of inflammation and oxidative stress markers (^·^O_2_^–^, H_2_O_2_, and ONOO^−^, ~30%) accompanied by an increase in NO bioavailability (100%). In agreement with the above findings by using the same technique, we observed in a similar magnitude an improvement of serum protein oxidation, mitochondrial dysfunction, DNA damage/repair, and apoptosis.

**Conclusion:**

In conclusion, we demonstrated that kefir improves cognitive deficits, which seems to be linked with three important factors of the AD—systemic inflammation, oxidative stress, and blood cell damage—and may be a promising adjuvant therapy against the AD progression.

## 1. Introduction

Alzheimer's disease (AD) is the most common cause of dementia in elderly patients and is clinically defined as a progressive, global, and strong cognitive decline leading to an emotional distress and codependence [1–4]. Unfortunately, the number of AD patients has been rapidly growing worldwide (increasing by 117% in the last 26 years), with the highest age standardized prevalence in Turkey and Brazil [5].

The pathophysiology of AD is multifactorial, involving microglial activation, excessive proinflammatory cytokines, vascular disorder, disrupted mitochondrial function accompanied by overproduction of reactive oxygen species (ROS), and oxidized molecules [6–13]. In this scenario, the cardinal neuropathological manifestations of AD culminate with amyloid-*β* (A*β*) and neurofibrillary tangles (NFTs) composed of hyperphosphorylated tau protein leading to synapse degeneration [9, 14, 15].

As the actual pharmacotherapy for dementia (using cholinesterase and/or glutamate inhibitors) is insufficient in slowing down completely the AD progression, new treatment strategies are still necessary to improve AD patient care [5, 16, 17]. In parallel, there is growing evidence that disturbances along the “brain-gut-microbiota axis” are involved in the pathogenesis of neurodegenerative diseases enhancing inflammation at the gut, systemic, and central nervous system (CNS) levels [15, 18]. In this context, recent data have shown the beneficial effects of probiotic supplementation on intestinal epithelial integrity, immunomodulation, oxidative stress, and even procognition [15, 19, 20]. However, the role of safe and inexpensive nutraceutical synbiotic kefir (a synergistic mixture of prebiotics and probiotics) in AD is still not documented [15, 20, 21].

The kefir-fermented milk is a functional food originally from the Northern Caucasus and currently distributed worldwide either commercially (e.g., Russia, Spain, Germany, United States, Canada, and Brazil) or “in-house” [22–26]. Milk fermentation with kefir grains is made up by bioactive compounds (peptides, vitamins, and polysaccharides) originally generated by acid lactic bacteria and yeast species present in these grains [23, 24, 26–28]. Recently, we and others have shown that administration of kefir and/or their bioproducts was able to prevent the cardiac and vascular dysfunctions in experimental models of hypertension [23, 26, 29], atherosclerosis [30], and gastric ulcers [27], which was justified, at least in part, by its antioxidative and immunomodulatory properties.

The present study addressed the hypothesis that kefir supplementation would provide cognitive benefit by attenuating systemic inflammation and oxidative stress in AD patients. The novel data revealed relevant new insights into the effects of this synbiotic on cognitive function through biochemical, molecular, and cellular parameters related to neurodegenerative diseases.

## 2. Methods

### 2.1. Patients

This uncontrolled clinical trial evaluated AD patients selected by convenience sampling. The diagnosis of AD was according to the clinical diagnostic criteria of dementia due to probable Alzheimer's disease with increased level of certainty defined by the Alzheimer's Association and the National Institute on Aging (NIA) published in 2011 [31]. The criteria evaluated in our study were based on the presence of insidious cognitive progressive decline or behavior symptoms involving a minimum of two cognitive domains (e.g., memory, language, attention, and constructive abilities) besides impairment of usual activities. It is important to emphasize that unexplained symptoms by delirium or major psychiatric disorders are detected through a combination of history taking and objective cognitive assessment. The sample included individuals of both sexes, without age restriction, who were assisted at a reference center in Vila Velha, Espírito Santo, Brazil, specialized in AD. The inclusion criteria were the following: (1) patients without previous neurological and/or psychiatric comorbidities associated with cognitive impairment, (2) patients without clinical depression and/or with a depression index < 17 using the Hamilton scale, and (3) patients without several clinical comorbidities (decompensated diabetes) and patients with autoimmune diseases taking or not immunosuppressive medication, neoplasms, and/or inflammatory bowel diseases. The exclusion criteria were the following: (1) patients unable to make use of probiotic supplementation because of organic or environmental causes, (2) patients using substances that might affect neurocognitive assessment, and (3) patients not using the maximum dose of acetylcholinesterase inhibitor (donepezil, 10 mg/day). The research was approved by the ethics committee of Vila Velha University (#1.804.392), and a signed free informed consent form was obtained from each of the subjects and/or their tutors after complete information about the nature and possible risks and benefits of the study, for him/herself, the community, and medical science was given.

**Figure 1:**
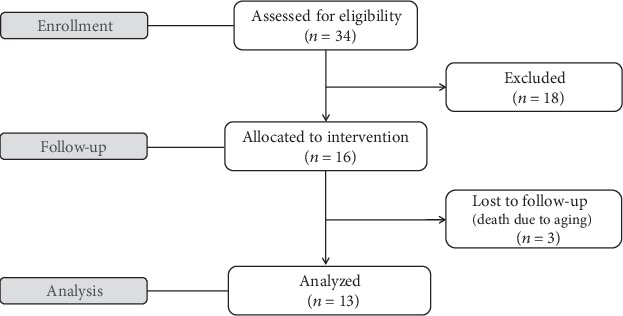
Summary of exclusion criteria used in the present study and patient flow.

In the beginning of the study, thirty-four subjects fulfilled the inclusion criteria. However, eighteen subjects were excluded from the study due to the elevated depression indexes (three subjects), due to clinical and laboratory signs of decompensated diabetes (six subjects), and due to the intervention with antibiotic therapy and/or hospitalization during the follow-up (nine subjects). During the study, 3 out of 16 patients in the final group died and a total of 13 subjects completed the experiments receiving the probiotic supplementation for 90 days and being evaluated, as summarized in Figure 1.

### 2.2. Production of Fermented Milk by Kefir Grains

The fermented material was prepared by inoculating pasteurized milk with 4% kefir grains containing the species *Acetobacter aceti*, *Acetobacter* sp., *Lactobacillus delbrueckii delbrueckii*, *Lactobacillus fermentum*, *Lactobacillus fructivorans*, *Enterococcus faecium*, *Leuconostoc* spp., *Lactobacillus kefiranofaciens*, *Candida famata*, and *Candida krusei* and incubating the culture at 25°C–28°C for 24 h. After the incubation period, the fermented product was filtered and refrigerated at 2°C–6°C for 24 h, as previously described by us [23]. In order to improve organoleptic characteristics, the product was blended with organic strawberries in the proportion of 500 g of fruit for every 2 L of fermented milk without added sugars or preservatives.

### 2.3. Experiment Protocol

At first (T0), the participants were submitted to a battery of tests for the screening of the identification of cognitive deficits and their venous blood was collected for analysis of inflammation, oxidative stress, and molecular and cellular integrity. The blood samples were collected in EDTA-containing Vacutainer glass tubes (Becton, Dickinson and Company, Franklin Lakes, NJ) and centrifuged at 2000 g for 10 min, and the serum was then stored at −20°C. In addition, erythrocytes were lysed and white blood cells were stored at -80°C. All the measurements were obtained via an automatic biochemical analyzer (AU 400 or 680, Olympus/Beckman Coulter, Munich, Germany) or a flow cytometer (FACSCanto II, BD, CA, USA). These data were considered the control values in this paired study. After, the patients were subjected to fermented milk supplementation at the daily dose of 2 mL per kilogram of body weight. At the end of the study (T90), the cognitive, biochemical, molecular, and cellular parameters were once again evaluated.

### 2.4. Cognitive Assessment

Cognitive assessment was made before (T0) and after 90 days (T90) of the probiotic supplementation, using the tests recommended by the Department of Cognitive Neurology and Aging of the Brazilian Society of Neurology for screening dementia syndromes [32], as well as by the American Society of Neurology [33] and the National Institute on Aging and Alzheimer's Association [34]. The following functions were analyzed: “global cognitive functions,” using the (1) Mini-Mental State Examination (MMSE); “memory,” using a recall board with 10 concrete objects to promote the (2) immediate memory test and (3) delayed memory test, according to Nitrini et al. [32], which is recommended for evaluating populations with different levels of education; “visual-spatial and abstraction abilities,” using the (4) Cookie Theft Picture Test, according to the consensus recommendations published by Nitrini et al. [32] suggesting the use of description of thematic figures justified by the absence of studies in the area with the Brazilian population, and the (5) Similarity Test, using the recommendations published by Nitrini et al. [32] to apply NEUROPSI subsection where the respondent is asked to say the similarity between three pairs of nouns (orange and pear, dog and horse, and eye and nose); “executive and language functions,” using the (6) Boston Naming Test and (7) verbal fluency test; “attentive function,” using (8) Trail Making Test A; and “visuoconstructive abilities,” using the (9) clock-drawing test. To avoid the *learning effect bias*, the cognitive assessment trials were spaced by 90 days and the various domain tests were applied on a different order.

### 2.5. Determination of Cytokines Using the Cytometric Bead Array

Concentrations of proinflammatory (IL-6, IL-8, IL-1b, IL-12p70, and TNF-*α*) and anti-inflammatory (IL-10) cytokines were analyzed in the serum of patients using a Cytometric Bead Array Human Inflammation kit (CBA, BD Biosciences, USA) according to the manufacturer's instructions. Samples were analyzed with a FACSCanto II flow cytometer (BD, San Jose, CA, USA). Data acquisition was performed with FACSDiva software (BD), and the analysis of the events acquired was performed with the help of FCAP Array software (BD). Samples were measured by comparing them with the standard curves of recombinant cytokines using FCAP Array software (BD). All results are expressed as pg/mL.

### 2.6. ROS Analysis

Quantification of ROS components was also performed by flow cytometry, using a FACSCanto II (Becton Dickinson, BD, CA, USA) instrument to analyze the intracytoplasmic ROS content, as previously described by us [27, 35, 36]. Peripheral blood was drawn from the Alzheimer's patients, and the red blood cell lysis was induced by the addition of ammonium chloride. Superoxide anion (^·^O_2_^−^), hydrogen peroxide (H_2_O_2_), peroxynitrite/hydroxyl radical (ONOO^−^/^·^OH^−^), and nitric oxide (^·^NO) were monitored separately by measuring changes in median fluorescence intensity (MFI) emitted by dihydroethidine (DHE), dichlorofluorescein (DCF), hydroxyphenyl fluorescein (HPF), and diaminofluorescein (DAF), respectively. Briefly, 10^6^ cells were incubated with 160 mmol/L of DHE, 20 mmol/L of DCF, 10 *μ*mol/L of HPF, or 2 *μ*mol/L of DAF at 37°C for 30 min (DHE, DCF, and HPF) or 180 min (DAF) in the dark. The samples were then washed, resuspended in PBS, and kept on ice until the acquisition of 10,000 events by flow cytometry, which were subsequently analyzed using FCS Express software (De Novo).

### 2.7. Advanced Oxidation Protein Products

To determine advanced oxidation protein products (AOPP), 40 *μ*L of plasma diluted in PBS (1.5) was added to 10 *μ*L of KI (1.16 mol/L) and 20 *μ*L glacial acetic acid and the absorbance was read at 340 nm in a microplate reader (Spectra-MAX-190, Molecular Devices, Sunnyvale, CA, USA). The formation of triiodide ion through the oxidation of KI with chloramine-T was used to quantify AOPP levels. Data are expressed as *μ*mol/mg of chloramine-T per mg of proteins, according to Witko-Sarsat et al. [37].

### 2.8. Mitochondrial Membrane Potential (MMP)

Estimation of mitochondrial membrane potential (MMP) was performed by flow cytometry using JC-10, a fluorogenic probe (Sigma-Aldrich, USA), following the manufacturer's instructions. Briefly, 2 × 10^6^ cells were loaded with 500 *μ*L of JC-10 solution at 37°C for 60 min, protected from light. For the positive control, cells were previously incubated with carbonyl cyanide 3-chlorophenyl-hydrazone (CCCP, 5 *μ*M), while unmarked cells were set as the negative control. JC-10 forms red J-aggregates in healthy cells but stays as a green monomer in cells that have lost mitochondrial integrity. The fluorescence intensities of JC-10 aggregates (red, FL2 channel) and monomers (green, FL1 channel) were measured with flow cytometer detectors and analyzed after compensation for spectral overlap. Data are expressed as the relative aggregate/monomer (FL2/FL1) ratio, which was assumed to be proportional to MMP intensity [38].

### 2.9. p53 and Cleaved PARP Expression

To determine the expression of p53 and cleaved PARP, 2 × 10^6^ cells were resuspended in Cytofix/Cytoperm (BD) solution and washed twice with Perm/Wash buffer (BD). Blood cells were separately incubated with 5 *μ*L of anti-p53-FITC (BD) or anticleaved PARP-PE, during 30 min in the dark. As the positive control, an aliquot of cells was treated with doxorubicin (25 *μ*g/mL, Sigma-Aldrich, USA) before antibody incubation. For the staining control, we used specific immunoglobulins (IgG) conjugated with FITC or PE. After antibody incubation, the samples were stained with 10 *μ*L of 7-amino-actinomycin D (7-AAD, BD). The determination of protein expression was acquired with the FACSCanto II flow cytometer (Becton Dickinson, San Jose, CA, USA) and analyzed using FCS Express software (De Novo). Data are expressed as the percentage of positive cells.

### 2.10. Cell Cycle Analysis

For the determination of cell cycle distribution, 2 × 10^6^ cells were resuspended in Cytofix/Cytoperm (BD) solution, washed with Perm/Wash buffer (BD), and incubated with 10 *μ*L of 7-amino-actinomycin D (7-AAD, BD) for 30 min, at 4°C, in the dark. The cell cycle profile was determined by the acquisition of 10,000 events per sample using the FACSCanto II flow cytometer for acquisition and the FCS Express software for analysis. The sample flow rate during acquisition did not exceed 200-300 cells per second. Data are expressed as the percentage of cells in each cell cycle phase, which are sub-G_0_-representing cells with fragmented DNA (DNA content < 2n), G_0_/G_1_-representing cells with 2n DNA, and S/G_2_/M-representing cells with DNA content > 2n [35, 36].

### 2.11. Cell Viability and Apoptosis

Apoptotic cells were identified and quantified by flow cytometry using the Annexin V-FITC/PI Apoptosis Detection kit (BD Bioscience) according to the manufacturer's instruction. Briefly, 10^6^ blood cells were resuspended in binding buffer and incubated for 15 min at room temperature, in the dark, with 5 *μ*L annexin V-FITC and 5 *μ*L propidium iodide (PI). Data were acquired using the FACSCanto II flow cytometer (BD) and analyzed by the FCS Express software (De Novo). Double-negative cells were considered viable, while annexin V-FITC-positive cells were considered apoptotic [27, 35, 36]. Data are expressed as percentage of cells.

### 2.12. Statistical Analysis

All data are expressed as the mean ± SEM (except for the characteristics of patients, expressed as means ± SD). The Kolmogorov-Smirnov test was applied to assess the normal distribution of data. Considering that all the samples had Gaussian distribution, Student's *t*-test for paired samples was used for the statistical analysis of cytokine concentration, ROS, apoptosis indexes, and cell viability before and after the administration of probiotic. *p* values < 0.05 were considered statistically significant. Statistical analysis was performed using the GraphPad Prism software, version 7.0.

## 3. Results

### 3.1. Characteristics of Patients (Demographic, Anthropometric, and Social Characteristics)

Table 1 shows the clinical characteristics of elderly patients included in this study. No significant differences between gender groups were observed in relation to age, body mass index (BMI), treatment duration, and education level.

### 3.2. Cognitive Assessment

**Figure 2:**
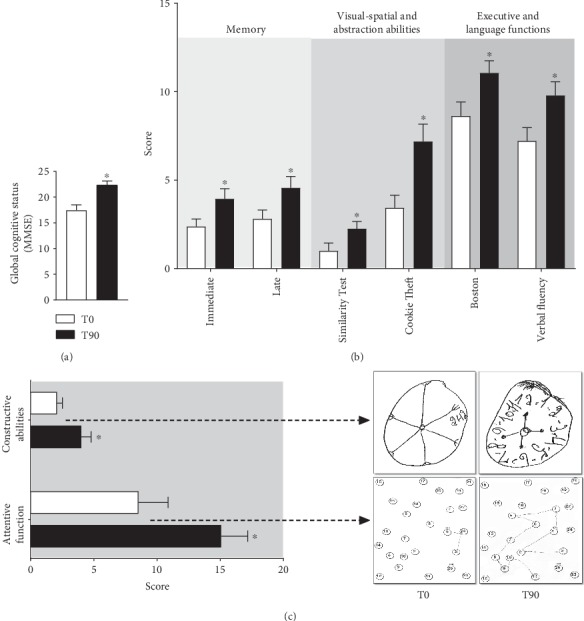
Series of panels showing the results of the evaluation of cognitive tests before and after treatment using kefir (a fermented milk with synbiotics). (a) Global cognitive status (by Mini-Mental State Examination (MMSE)) comparing the effect of the treatment with the previous observed values. (b) Memory analysis (by immediate and late tests, left panel), visual-spatial and abstraction abilities (by Similarity Test and Cookie Theft Picture Test, center panel), and executive and language functions (by Boston Naming Test and verbal fluency test, right panel). (c) Cognitive battery assay, evaluated through constructive abilities and attentive function, with typical hand drawings of the patients during the applied test. The results are expressed as mean ± SEM (*n* = 13). ^∗^*p* < 0.05 compared to T0.

Figure 2 summarizes the results of the cognitive tests divided into T0 and T90 time points. We observed an improvement of performance in the MMSE in 28% (T0: 17.4 ± 1.03 hits and T90: 22.3 ± 0.82 hits, *p* < 0.0001), indicating the benefits of kefir-fermented milk on “global cognitive status” (Figure 2(a)). A similar impact was observed on “memory analysis” (Figure 2(b), left panel) through the immediate memory test (~66%, *p* < 0.05) and late memory test (~62%, *p* < 0.05) between T0 and T90. In the center of Figure 2(b), we also demonstrate the improvement of “visual-spatial and abstraction abilities” using the Similarity Test (~2-fold increase, *p* < 0.05) and Cookie Theft Picture Test (~2-fold increase, *p* < 0.05). Concerning the “executive and language functions” (Figure 2(b), right panel), we also note a significant increment through the Boston Test (~30%, *p* < 0.05) and verbal fluency test (~25%, *p* < 0.05). Finally, the “cognitive battery assay” showed a significant amelioration on the constructive abilities, evidenced by the improvement on the clock-drawing test (T0: 9.0 ± 2.4 hits and T90: 13.2 ± 2.3 hits, *p* < 0.05), and on attentive function testified by the Trail Making Test (40%, *p* < 0.05).

### 3.3. Cytokines

**Figure 3:**
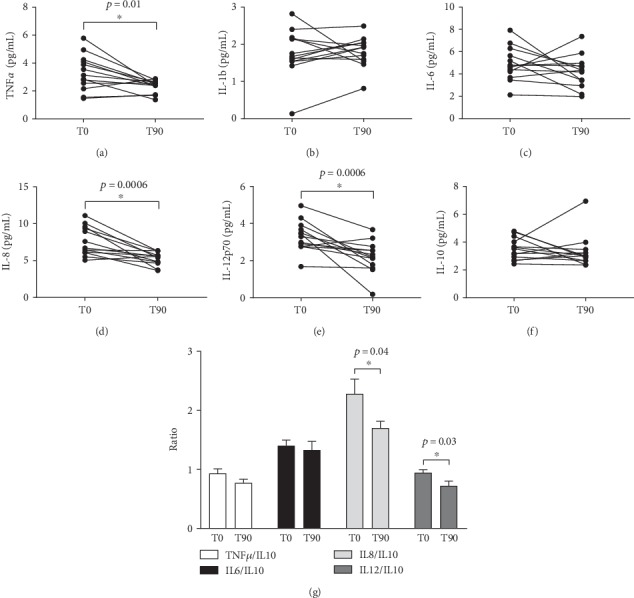
Cytokine levels measured by the Cytometric Bead Array (CBA) using protocols performed through flow cytometry analysis. TNF-*α* (a), IL-1b (b), IL-6 (c), IL-8 (d), IL-12p70 (e), IL-10 (f), and the ratio of proinflammatory/anti-inflammatory markers (g) were measured before and after probiotic supplementation. The results are expressed as mean ± SEM (*n* = 13). ^∗^*p* < 0.05 compared to T0.

Figure 3 shows the quantification of some cytokines involved in pathogenesis of neurodegenerative diseases. The levels of proinflammatory cytokines TNF-*α*, IL-8, and IL12p70 were lower at T90 than at T0 (~1.5-fold decrease, respectively). However, other proinflammatory (such as IL-1b and IL-6) or anti-inflammatory (IL-10) cytokines did not show difference between T90 and T0. Interestingly, analyzing the balance between proinflammatory and anti-inflammatory cytokines (Figure 3(g)), we verified that the probiotic supplementation was able to reduce the IL-8/IL-10 and IL-12/IL-10 ratios (T0: 2.3 ± 0.2 vs. T90: 1.7 ± 0.1 pg/mL and T0 : 0.95 ± 0.05 vs. T90: 0.72 ± 0.08 pg/mL, respectively, *p* < 0.05).

### 3.4. Direct and Indirect Oxidative Stress Biomarkers

**Figure 4:**
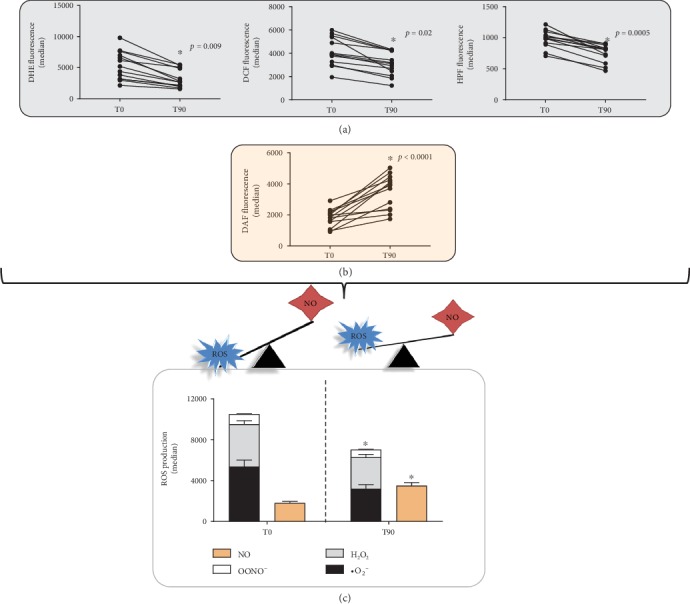
Superoxide anion, hydrogen peroxide, peroxynitrite/hydroxyl radical, and nitric oxide levels measured by specific biomarkers commonly used to evaluate ROS (DHE, DCF, HPF, and DAF staining, respectively). Records of ROS production were made before and after 90 days of the probiotic supplementation. Note a marked recovery of the ROS imbalance after the treatment. The results were expressed as mean ± SEM (*n* = 13). ^∗^*p* < 0.05 compared to T0.

Oxidative stress was evaluated by flow cytometry trough DHE, DCF, HPF, and DAF fluorescence.  Figure 4 shows systemic ROS production measured before and after kefir treatment. We observed a significant decrease in serum levels of ^·^O_2_^−^, H_2_O_2_, and ONOO^−^/OH^−^ (Figure 4(a)) with a simultaneous increase in NO levels (Figure 4(b)). The bar graph in Figure 4(c) represents the mean values of systemic ^·^O_2_^−^ (T0: 5953 ± 999 vs. T90: 3622 ± 707, a.u.), H_2_O_2_ (T0: 4580 ± 611 vs. T90: 3202 ± 286, a.u.), ONOO^−^/^·^OH^−^ (T0: 1161 ± 70 vs. T90: 874 ± 34, a.u.), and NO (T0: 3493 ± 304 vs. T90: 1799 ± 158) between T0 and T90.

**Figure 5:**
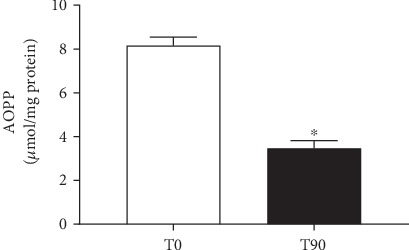
Levels of advanced oxidative protein products before and after probiotic supplementation. The results are expressed as mean ± SEM (*n* = 13). ^∗^*p* < 0.05 compared to T0.

Figure 5 shows the assessment of systemic protein oxidation by AOPP (an important indirect biomarker of oxidative stress). The results revealed that kefir administration leads to a remarkable decrease in protein oxidation (T0: 8.4 ± 0.6 vs. T90: 2.9 ± 0.3 *μ*mol/mg).

### 3.5. Mitochondrial Membrane Potential (MMP)

**Figure 6:**
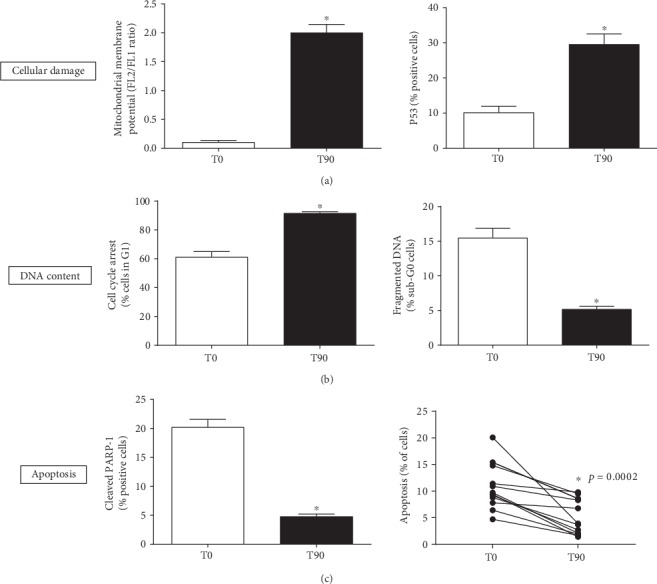
Different evidences that kefir supplementation protects against cellular damage. The mitochondrial membrane potential (MMP) and p53 expression (a), followed by induced cell cycle arrest and DNA repair (b), accompanied by a marked decrease in apoptosis (c). The results are expressed as mean ± SEM (*n* = 13). ^∗^*p* < 0.05 compared to T0.

We further evaluated whether the decrease in ROS production was accompanied by a recovery of mitochondrial membrane potential (MMP) due to kefir administration. The level of membrane polarization after kefir treatment is shown in Figure 6(a) (left panel). JC-10 green fluorescence (reflecting mitochondrial dysfunction) significantly decreased, and JC-10 red fluorescence (reflecting mitochondrial integrity) increased after kefir consumption. Most of the cells shifted towards the red fluorescence after kefir consumption indicating a significant preservation of mitochondrial function (T0: 0.11 ± 0.03 vs. T90: 2.0 ± 0.14, FL2/FL1, a.u.).

### 3.6. p53 Expression

The protein p53 is a transcription factor that plays an important role in maintaining the genome integrity by controlling cell apoptosis and cell cycle arrest through signaling of genotoxic stress, like oxidative stress. Figure 6(a) (right panel) shows the values of p53 expression levels measured in the blood samples before and after kefir treatment. As shown, p53 expression increased from T0 (10.1 ± 1.8%) to T90 (29.6 ± 2.9%).

### 3.7. Cell Cycle Arrest

Cell cycle distribution was determined using flow cytometric analysis of blood cells. As shown in Figure 6(b) (left panel), kefir consumption induced an increase in the G_0_/G_1_ phase, indicating a cell cycle arrest in T90 in comparison to T0 (T0: 61 ± 3.7 vs. T90: 92 ± 1.0%). Simultaneously, we observed a decrease in the percentage of cells in S/G_2_/M phases of the cell cycle after kefir consumption (data not shown).

### 3.8. DNA Fragmentation

Results showed a significant decrease in cells with sub-G_0_ DNA content after kefir treatment. A DNA fragmentation assay was performed to determine whether kefir was capable of protecting DNA from damage in cells undergoing increased ROS production. As shown in Figure 6(b) (right panel), kefir administration was able to reduce DNA fragmentation from 15.5 ± 1.3% in T0 to 5.2 ± 0.4% in T90. This finding suggests significant changes in cell cycle progression and induction of apoptosis.

### 3.9. Cleavage of PARP-1

Poly (ADP-ribose) polymerase 1 (PARP-1) is involved in several biological processes, such as cell cycle progression, DNA repair and regulation of transcription, and programmed cell death. Proteolytic cleavage of PARP is considered a hallmark of apoptosis, since PARP-1 is cleaved by activated apoptotic caspases. Our flow cytometry analysis showed a significant reduction in the percentage of cleaved PARP-1 after kefir consumption (T0: 20.2 ± 1.3 vs. T90: 4.8 ± 0.4%) (Figure 6(c), left panel).

### 3.10. Apoptosis Assay

Flow cytometry was used to determine the antiapoptotic effect of kefir by quantification of annexin V-positive cells. Phosphatidylserine (PS) is externalized and available for detection by annexin V when cells undergo apoptosis. After kefir treatment, the percentage of annexin V-positive cells decreased in T90 (6.86 ± 1.91%) compared to T0 (12.88 ± 1.91%) (Figure 6(c), right panel); consequently, majority of cells were negative for annexin V indicating their healthy status. The viable-to-apoptotic cell ratio (V/A ratio) was 2.05, and the mean V/A ratio increased from 6.53 at T0 to 13.39 at T90.

## 4. Discussion

In 1908, a Russian zoologist named Metchnikoff (the Nobel laureate who discovered phagocytosis) popularized for the first time the consumption of probiotics in the form of yogurt as a “healthy food” [39–41]. However, only 100 years later, it has been recognized that probiotics may influence CNS function via the microbiota-gut-brain axis [19, 42, 43]. Even so, clinical investigations using probiotics in elderly patients with dementia are still scarce in medical literature [15]. In this context, our study is the first to evaluate the beneficial effects of kefir supplementation (at the minimum dose of 2 mL/kg for 90 days) on cognitive function, biomarkers of systemic oxidative stress, inflammation, and cell damage in elderly patients with AD.

Although it is known that AD patients are susceptible to multiple complications related to cognitive performance, the innovative therapeutic strategies to reverse this progression are still scant. Interestingly, our results with kefir supplementation for 3 months improved all cognitive tests applied in our experiment (memory, visual-spatial function and abstraction abilities, executive and language functions, constructive abilities, and attentive function). Our results corroborate the findings of Akbari et al. [19] that also demonstrated that probiotic milk (containing *Lactobacillus acidophilus*, *Lactobacillus casei*, *Bifidobacterium bifidum*, and *Lactobacillus fermentum*) for 12 weeks are able to improve the cognitive function. More recently, Kobayashi et al. [44] reported that oral 24-week supplementation with *Bifidobacterium breve* A1 improved cognitive function in AD patients. As suggested by others, we speculated that kefir supplementation also could alter the gut microbiota contributing to neuromodulation through neuroactive and neuroendocrine synthesis (e.g., acetylcholine, dopamine, serotonin, norepinephrine, adrenaline, glutamate, gamma-aminobutyric acid, and brain-derived neurotrophic factor (BDNF)) besides their related receptor expression [15, 19, 45–49]. This hypothesis is partially based on early findings in AD patients using multispecies probiotic intake (for 1 month) improving gut bacteria composition and serum tryptophan levels, an amino acid precursor in serotonin and melatonin biosynthesis [50]. Moreover, experimental evidences support this idea demonstrating decreased level of serum serotonin, BDNF, and NMDA receptors in germ-free mice compared to conventional mice [19, 51, 52] and that *Lactobacilli* supplementation can increase GABA availability from glutamate [53]. More recently, another data demonstrated in mice revealed that the gut microbiota is a potent influencer of BDNF in cortical and hippocampal areas, besides increasing striatal monoamine turnover and modulating the expression of serotonin receptor 1A [48, 54]. Despite all these neuromodulatory benefits previously described, it is believed that the influence on inflammation and oxidative stress by probiotics may also contribute to the neuroprotective effect, thus justifying the next step of this study.

Neuroinflammation has been observed as another relevant player in AD pathogenesis in both experimental and clinical studies [11, 12, 55–59]. Numerous data demonstrate positive associations between proinflammatory cytokines (e.g., IL-1, IL-6, TNF-*α*, IL-8, and IL-12) and the progression of AD [58, 60]. Moreover, recent investigations reported that these neuroinflammatory cytokines can compromise the clearance of A*β*, accumulating this toxic protein in the brain [57, 58, 61–63]. Thus, cytokine balance has been an important research target for understanding the pathophysiology of AD and identifying new potential therapeutic targets. At the same time, emerging data have shown that probiotics can secrete metabolites and factors with immunomodulatory properties [24, 48, 64]. For example, relevant studies demonstrated a decrease in proinflammatory cytokines using multistrain probiotic supplementation [21, 65, 66]. The novelty in our study was that we used an inexpensive food (and easily produced at home) to reduce serum proinflammatory cytokines and possibly contribute to neuroprotective effect in AD patients. This immunosuppressive profile is reinforced by other recent study from our lab using only nonbacterial fraction of kefir in dyslipidemic mice [30], which allows us to speculate that this immunomodulation could be the result of a synergistic effect between microorganisms and soluble products present in kefir.

Increased levels of serum oxidative stress biomarkers reported in neurodegenerative diseases [11, 19, 67–71] seem to be an interesting approach to evaluate the impact of new therapeutic strategies in AD patients. At the same time, there is a strong correlation of antioxidant-rich diets as an “easy” strategy for neuroprotection [11, 17, 72, 73], including probiotics [19, 21]. In this study, the assessment of serum oxidative stress by direct and indirect methods (ROS and AOPP) demonstrates that kefir has significant antioxidant effects, helping to explain the favorable result concerning cognition in AD patients. It is known that excessive ROS are involved as a cause and consequence of proinflammation contributing directly and indirectly to the pathogenesis of AD [8, 17, 74–76]. More specifically, due to their electronegativity and reactivity, ^·^O_2_^−^, H_2_O_2_, and ONOO^−^/^·^OH^−^ can oxidize lipids, nucleic acids, and proteins leading to mitochondrial destruction, neuroexcitotoxicity (e.g., excessive Ca^2+^ influx and formation of aggregates of toxic proteins), and stimulation of microglia and astrocytes to develop inflammatory response [17, 24, 36, 77]. On the other hand, NO (by eNOS or nNOS isoforms) seems to be a “ROS-gasotransmitter” with important neuroprotective properties such as antioxidant (acting as a scavenger of ^·^O_2_^−^), vasodilator (increasing cerebral blood supply to neurons), inhibitor of NMDA receptors at glutamatergic synapses (thereby preventing neuroexcitotoxicity), and preventing the deposition of A*β* [10, 78–81]. Interestingly, our findings demonstrate for the first time that kefir reduces serum ROS bioavailability accompanied by an NO increase reflecting the reduction of plasma protein oxidation in AD patients. These data may be supported by experiments of Musa et al. [21] that revealed an increase in the activity of antioxidant enzymes (SOD, GSH, and GPx) in the brain tissue of mice treated with *Lactobacillus*. Therefore, all these results motivated us to investigate the impact of mitochondrial and cell damage obtained by the same blood samples from the same subjects.

**Figure 7:**
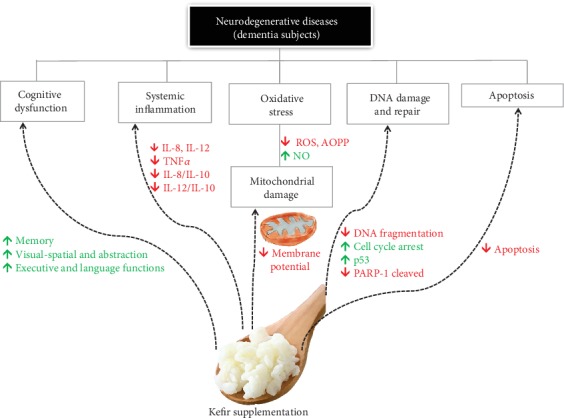
Beneficial effects of kefir on dementia in AD patients. Simplified scheme of main effects after 90 days with kefir supplementation on Alzheimer's subjects.

It is known that AD brain mitochondria develop diminished membrane potential, disrupting the electron transfer chain, favoring excess ROS production, alteration in cytosolic calcium homeostasis, and A*β* accumulation leading to neurodegeneration [11, 72, 82–85]. In parallel, Delbarba et al. [86] demonstrated that mitochondrial DNA can decrease in peripheral blood in the early stages of neurodegenerative disease progression, being a potential “blood-based signature” in AD patients. Therefore, since several studies of mitochondrial damage in Alzheimer's patients are performed postmortem, the possibility of exploring blood samples in clinical investigations (as in the present study) may be a promising strategy for establishing mechanisms related to cognitive improvement with immunomodulatory, antioxidant, and/or mitochondrial function. Interestingly, we observed that kefir was able to reverse the compromised mitochondrial membrane potential in blood cells of AD patients, helping to explain (at least in part) the reduction of ROS bioavailability in the same patients. Furthermore, in this same context, as it is known that p53 under lower ROS levels can contribute to cellular survival [87–89], we confirmed this protective effect by kefir in AD patients through 2 evidences: (1) by induction of DNA repair and (2) by reduction of apoptosis. Firstly, we may affirm this beneficial effect through a decrease in DNA fragmentation accompanied by induction of cell cycle arrest after 90 days of supplementation. Secondly, the reduction of apoptosis by kefir was detected through a reduction of cleaved PARP-1 (considered to be a classical hallmark of apoptosis) and by a decrease in annexin V-positive cells [36, 90]. In summary, our data indicate that kefir supplementation has also a mitochondria-protective effect in addition to cytoprotective and antiapoptotic action, whose effects try to mitigate the progression of neurodegeneration (Figure 7).

This study had some potential methodological limitations. First, due to difficulties in obtaining fresh fecal samples, we had problems with analysis of the fecal microbiota before and after the kefir supplementation in our AD patients. Second, our study was conducted without control participants using other type of fermented milk. Third, the sample size was small, but justified by age of the patients, and it was accompanied by severe exclusion criteria. Additionally, the impact of learning effect bias was minimized through randomized procedures applied in the cognitive tests in the present study. Lastly, but not least, we could have enriched the data exploring imaging biomarkers (e.g., magnetic resonance imaging and PET) or specific biomarkers (e.g., brain-derived neurotrophic factor, neuronal butyrylcholinesterase, and apolipoprotein A1) which would extend the possible beneficial effects induced by chronic kefir administration in these AD patients. Despite these limitations, the novelty of our study is to demonstrate the beneficial effects of chronic kefir supplementation on the cognitive function in the elderly with AD.

## 5. Conclusion

The current study demonstrated that synbiotic supplementation for 90 days to older patients with AD had reparatory favorable effects on cognitive dysfunction (improving memory, language, executive functions, visual-spatial function, conceptualization, and abstraction abilities), systemic inflammation (by reduction of proinflammatory cytokines), systemic oxidative stress (verified by a decrease in ROS and AOPP), and blood cell damage (analyzed by DNA damage/repairment and apoptosis). Therefore, the data of the present study is opening a great opportunity for the evaluation of the clinical benefits of probiotics/synbiotics at larger randomized controlled clinical trials, strengthening the present valuable findings.

## Figures and Tables

**Table 1:** Patient characteristics.

Parameters	Women (*n* = 11)	Men (*n* = 2)	*p* value
Age (years)	78.7 ± 3	78 ± 7	0.93
BMI (kg/m^2^)	25.8 ± 0.6	27 ± 1.7	0.61
Education level (years)	5.9 ± 0.6	5 ± 1.0	0.51
Treatment duration (years)	1.85 ± 0.7	0.6 ± 0.1	0.08

^∗^The values are presented as mean ± SD.

## Data Availability

All data used to support the findings of this study are included within the article.

## Abbreviations

DA: Alzheimer's disease
CNS: Central nervous system
ROS: Reactive oxygen species
MMSE: Mini-Mental State Examination
CBA: Cytometric Bead Array
MFI: Median fluorescence intensity
DHE: Dihydroethidine
DCF: Dichlorofluorescein
HPF: Hydroxyphenyl fluorescein
DAF: Diaminofluorescein
AOPP: Advanced oxidation protein products
MMP: Mitochondrial membrane potential
CCCP: Carbonyl cyanide 3-chlorophenyl-hydrazone
PI: Propidium iodide
BMI: Body mass index
PARP-1: Poly (ADP-ribose) polymerase 1
PS: Phosphatidylserine
V/A ratio: Viable-to-apoptotic cell ratio
BDNF: Brain-derived neurotrophic factor
SEM: Standard error of the mean
UVV: Vila Velha University.
